# Quantifying sedation satisfaction during bronchoscopy using the Bispectral Index

**DOI:** 10.1186/cc13610

**Published:** 2014-03-17

**Authors:** J Palminteri, J Dziodzio, D Seder, J Zuckerman, R Riker

**Affiliations:** 1 Maine Medical Center, Portland, ME, USA

## Introduction

Sedation monitoring with the Bispectral Index (BIS) is uncommon during bronchoscopy but allows for a more objective measure of sedation; we hypothesized that higher BIS scores would correlate with lower patient satisfaction and that lower BIS scores (deep sedation) would result in better patient and physician satisfaction.

## Methods

Between October 2012 and August 2013, bronchoscopy using conscious (CS) or monitored anesthetic deep sedation (DS) was monitored with BIS. Medication administration and procedures were time-stamped. Sedation was administered blinded to BIS score. DS cases used propofol infusions, CS cases used bolus midazolam and fentanyl. Providers rated sedation satisfaction, cough, and the ability to perform the intended procedure using a 100 mm visual analog scale at the end of the procedure blind to BIS (0 unsatisfied to 100 satisfied). Patients were surveyed at 1 hour and 24 hours regarding overall sedation, symptoms, and procedure recall (unpleasant recall 1, no recall 4). Group differences were considered statistically significant at *P *< 0.05.

## Results

Twenty-six procedures were monitored, 20 with CS and six endobronchial ultrasound procedures with DS. There was no difference with respect to age or gender. The mean doses of midazolam and fentanyl were 5 mg and 85 μg, respectively. BIS values were lower at all predefined points of the procedure for DS cases versus CS. Physicians were more satisfied with sedation and the lack of cough with DS, but there was no significant difference in patient satisfaction between the two groups with regards to overall sedation, procedure-related symptoms or willingness to have repeat bronchoscopy. Patients with no recall had lower nadir BIS scores (46 vs. 71, *P *= 0.03) and scores at procedure end (76 vs. 94, *P *= 0.04) compared with those with any recall. There was no difference in the doses of midazolam or fentanyl in CS cases despite statistically significant differences in patient recall and BIS scores. Junior fellows scored greater satisfaction with sedation, were less bothered by cough and more often felt able to perform the intended procedure compared with senior fellows.

## Conclusion

Deep sedation resulted in greater physician satisfaction with procedural conditions as well as lower BIS scores but no significant difference in patient satisfaction compared with conscious sedation. Patients with no recall of the procedure had lower BIS scores at the nadir and end of the procedure. BIS may be a novel tool to monitor procedural depth, allowing proceduralists to better monitor the narrow window between adequate sedation and dangerous oversedation.

